# Biochanin A, a Phytoestrogenic Isoflavone with Selective Inhibition of Phosphodiesterase 4, Suppresses Ovalbumin-Induced Airway Hyperresponsiveness

**DOI:** 10.1155/2011/635058

**Published:** 2011-03-14

**Authors:** Wun-Chang Ko, Ling-Hung Lin, Hsin-Yi Shen, Chi-Yin Lai, Chien-Ming Chen, Chung-Hung Shih

**Affiliations:** ^1^Department of Pharmacology, School of Medicine, College of Medicine, Taipei Medical University, Taipei 110, Taiwan; ^2^Department of Otolaryngology, Taipei Medical University Hospital, Taipei 110, Taiwan; ^3^Division of Endodontics, Department of Dentistry, Taipei Medical University Hospital, Taipei 110, Taiwan; ^4^Department of Medical Technology, School of Medicine, College of Medicine, Taipei Medical University, Taipei 110, Taiwan; ^5^School of Respiratory Therapy, College of Medicine, Taipei Medical University, 250 WuXing Street, Taipei 110, Taiwan

## Abstract

The present study investigated the potential of biochanin A, a phytoestrogenic isoflavone of red clover (*Triflolium pratense*), for use in treating asthma or chronic obstructive pulmonary disease (COPD). Biochanin A (100 *μ*mol/kg, orally (p.o.)) significantly attenuated airway resistance (*R*_*L*_), enhanced pause (*P*_enh_), and increased lung dynamic compliance (*C*_dyn_) values induced by methacholine (MCh) in sensitized and challenged mice. It also significantly suppressed an increase in the number of total inflammatory cells, neutrophils, and eosinophils, and levels of cytokines, including interleukin (IL)-2, IL-4, IL-5, and tumor necrosis factor (TNF)-*α* in bronchoalveolar lavage fluid (BALF) of the mice. However, it did not influence interferon (IFN)-*γ* levels. Biochanin A (100 *μ*mol/kg, p.o.) also significantly suppressed the total and ovalbumin (OVA)-specific immunoglobulin E (IgE) levels in the serum and BALF, and enhanced the total IgG_2a_ level in the serum of these mice. The PDE4_H_/PDE4_L_ value of biochanin A was calculated as >35. Biochanin A did not influence xylazine/ketamine-induced anesthesia. Biochanin A (10~30 *μ*M) significantly reduced cumulative OVA (10~100 *μ*g/mL)-induced contractions in the isolated guinea pig trachealis, suggesting that it inhibits degranulation of mast cells. In conclusion, red clover containing biochanin A has the potential for treating allergic asthma and COPD.

## 1. Introduction

Red clover (*Triflolium pratense* L., Fabaceae) contains formononetin (4′-*O*-methyldaidzein), biochanin A (4′-*O*-methylgenistein), daidzein, and genistein in relative proportions of 5.46%, 1.97%, 0.43%, and 0.11% [1]. These phytoestrogenic isoflavones are also present in soy; however, the contents of formononetin and biochanin A are substantially higher in red clover [2]. Since the 19th century, red clover tea or tincture has been used in North America as an antispasmodic for whooping cough, measles, bronchitis, laryngitis, and tuberculosis [3]. According to an early edition of the National Formulary, red clover has also been used as a treatment for asthma. However, the efficacy of most of the above-mentioned traditional red clover treatments has not been tested in randomized, placebo-controlled clinical studies [2]. Recently, botanical dietary supplements containing red clover have received a great deal of attention for treating the symptoms of menopause, the maintenance/improvement of bone, and cardiovascular health. It has also been reported to have a benign effect on breasts and the endometrium [2]. In addition, estrogen receptor-targeted therapeutics have proven successful in treating breast cancer and metabolic disorders, since phytoestrogens were reported as an estrogen-related receptor *α* agonist [4]. The cancer-protective effects of flavonoids are attributed to a wide variety of mechanisms, including modulating enzyme activity resulting in a decrease in the carcinogenicity of xenobiotics. A number of naturally occurring flavonoids have been shown to modulate the cytochrome P450 (CYP) enzyme system, including the induction of specific CYP isozymes, and the activation or inhibition of these enzymes. Isoflavones inhibit the activity of aromatase (CYP19), thus decreasing estrogen biosynthesis and producing antiestrogenic effects, important in the treatment of breast and prostate cancer [5]. In 2004, we reported that biochanin A selectively inhibited phosphodiesterase (PDE)4 activity with an IC_50_ value of 8.5 *μ*M, although PDE1 (IC_50_, 29.1 *μ*M) and PDE2 (IC_50_, 27.9 *μ*M) activities were also inhibited by the compound. However, biochanin A did not inhibit (IC_50_ > 100 *μ*M) PDE3 or PDE5 activities [6]. Whether formononetin inhibits PDE4 activity remains unknown. In our previous report, genistein and daidzein selectively inhibited PDE2 and PDE3 activities, respectively [6].

PDEs are classified according to primary protein and complementary (c)DNA sequences, cofactors, substrate specificities, and pharmacological roles. It is now known that PDEs comprise at least 11 distinct enzyme families hydrolyzing cAMP and/or cGMP [7]. PDE1~5 isozymes, which are calcium/calmodulin-dependent (PDE1), cGMP-stimulated (PDE2), cGMP-inhibited (PDE3), cAMP-specific (PDE4), and cGMP-specific (PDE5) were found to be present in the canine trachea [8], guinea pig lung [9], and human bronchi [10]. PDE3 and PDE4 were identified in the guinea pig airway [11], but other isozymes might also be present. Rolipram, a prototype PDE4 selective inhibitor, has both a high (PDE4_H_) and low (PDE4_L_) affinity for PDE4. In general, it is believed that the inhibition of PDE4_H_ is associated with adverse responses, such as nausea, vomiting, and gastric hypersecretion, and that the inhibition of PDE4_L_ is associated with anti-inflammatory and bronchodilating effects. Therefore the therapeutic ratio of selective PDE4 inhibitors for use in treating asthma and chronic obstructive pulmonary disease (COPD) is defined as the PDE4_H_/PDE4_L_ ratio [12]. In this study, biochanin A showed a higher PDE4_H_/PDE4_L_ ratio (>35) than the selective PDE4 inhibitor AWD 12-281 (11) [13]. The aim of the present study was to determine the potential of biochanin A, contained in red clover, in treating asthma or COPD.

## 2. Methods

### 2.1. Reagents and Animals

Biochanin A (mol wt., 284.27), ovalbumin (OVA), methacholine (MCh), polyethyleneimine, aluminum sulfate hexadecahydrate, dimethylsulfoxide (DMSO), bis(2-hydroxyethyl)aminotris(hydroxymethyl)methane (Bis-Tris), chloralose, urethane, Tris-HCl, benzamidine, phenylmethanesulfonyl fluoride (PMSF), Tween 20, *d,l*-dithiothreitol, ethylenediaminetetraacetic acid (EDTA), xylazine, and ketamine were purchased from Sigma Chemical (St. Louis, MO, USA). Rolipram and 4-(3-butoxy-4-methoxybenzyl)-2-imidazolidinone (Ro 20-1724) were purchased from Biomol (Plymouth Meeting, PA, USA). Freund's complete adjuvant (*Mycobacterium butyricum*) was purchased from Pierce Biotechnology (Rockford, IL, USA). Mouse T helper (Th)1/Th2 cytokine CBA kits, and mouse IgE and IgG_2a_ ELISA sets were purchased from Pharmingen (San Diego, CA, USA). Polyethyleneglycol (PEG) 400, and ethyl alcohol were purchased from Merck (Darmstadt, Germany). [Methyl-^3^H]-Rolipram was purchased from Amersham Pharmacia Biotech (Buckinghamshire, UK). Other reagents, such as CaCl_2_, MgCl_2_, and NaCl, were of analytical grade. Biochanin A, rolipram, and Ro 20-1724 were dissolved in a mixture of ethyl alcohol and DMSO (1 : 1). Other drugs were dissolved in distilled water. The final concentration of ethyl alcohol or DMSO *in vitro* was ≤0.5%, and did not significantly influence tracheal contractions or the binding of test drugs to high-affinity rolipram binding sites (HARBSs).

Male Hartley guinea pigs (500~600 g) and female BABL/c mice at age 8–12 weeks were obtained from the Animal Center of the National Science Council (Taipei, Taiwan). The animals were housed in ordinary cages at 22 ± 1°C with a humidity of 50%~60% under a constant 12/12-h light/dark cycle and provided with food and water *ad libitum*. Under a protocol approved by the Animal Care and Use Committee of Taipei Medical University, the following *in vivo* and *in vitro* experiments were performed.

### 2.2. Determination of PDE4_H_ Values

After the above-described guinea pigs were sacrificed, the whole brains were removed and homogenized with a glass/Teflon homogenizer (Glas-Col, Terre Haute, IN, USA) in 10 volumes of cold medium (pH 6.5) containing 20 mM Bis-Tris, 2 mM benzamidine, 2 mM EDTA, 50 mM sodium chloride, 0.1 mM PMSF, and 1 mM dithiothreitol. At 4°C, the homogenate was centrifuged at 170 g for 5 min to remove connective tissues and blood vessels. The suspended homogenate was then recentrifuged at 40,000 g for 30 min to separate the cytosolic and particulate portions. After washing three times with homogenizing buffer, the particulate portion was resuspended at a concentration of 400 mg/mL (wet weight/volume). The particulate portion mainly consisted of cell membranes. The ability of biochanin A (10~300 *μ*M) or rolipram (0.1~1000 nM), a reference drug, to bind to HARBSs of the membranes was determined by replacing 2 nM [^3^H]-rolipram in a reaction buffer at 30°C for 1 h, according to a modified version of method described by previous investigators [14, 15]. Briefly, the reaction buffer consisted of 50 mM Tris-HCl and 5 mM MgCl_2_ (pH 7.5). The total volume of the reaction mixture was 25 *μ*L, consisting of 10 *μ*L of particulate suspension, 10 *μ*L of [^3^ *H*]-rolipram, and 5 *μ*L of biochanin A or rolipram. After 1 h, the reaction was terminated by placing the reaction vessel in crushed ice. The reaction mixture was then transferred onto Whatman GF/B glass-fiber filters soaked in a 0.3% polyethyleneimine solution in a minifunnel. The reaction mixture was filtered by centrifuging it at 90 g for 10 s and the filtrate was collected in a 1.5-mL Eppendorf tube with a top adapted to fit the outlet of the minifunnel. The filters were each washed three times with 300 *μ*L of reaction buffer in the same manner and transferred into a 2 mL cocktail for radiation counting (total binding) using a *β*-scintillation counter (Beckman, Fullerton, CA, USA). Nonspecific binding, which was defined in the presence of 10 *μ*M Ro 20-1724, was subtracted from the total binding to yield the specific binding. The effective concentrations (EC_50_) values of biochanin A and rolipram, at which half of the [^3^ *H*]-rolipram bound with HARBSs of cell membranes was displaced, were defined as the PDE4_H_ values and these may be correlated with adverse effects, such as nausea, vomiting, and gastric hypersecretion [16].

### 2.3. Airway Hyperresponsiveness In Vivo

Ten female BABL/c mice in each group were sensitized on days 0 and 14 by an intraperitoneal (i.p.) injection of 20 *μ*g of OVA emulsified in 2.25 mg aluminum hydroxide gel, prepared from aluminum sulfate hexadecahydrate, in a total volume of 100 *μ*L. On day 21, these mice were injected with (i.p.) 100 *μ*L of a mixture of 1% OVA and Freund's complete adjuvant (1 : 1). Mice were challenged *via* the airway on days 28, 29, and 30 by ultrasonic nebulization using 1% OVA in saline for 30 min. After the last of the primary OVA challenges [17], airway hyperresponsiveness (AHR) was assessed on day 32 (48 h after 1% OVA provocation) in each group. Each group of mice was orally (p.o.) administered a vehicle (control) or 30~100 *μ*mol/kg of biochanin A 2 h before and 6 and 24 h after OVA provocation. For comparison, sham-treated mice were sensitized but challenged with saline instead of 1% OVA (nonchallenged). The vehicle, a mixture of alcohol : DMSO : Tween 20 : saline (0.5 : 0.5 : 1 : 8, v/v) or biochanin A was orally administered at a volume of 0.01 mL/g of body weight. AHR was assessed using two methods: (1) in anesthetized ventilated mice, AHR was assessed as previously described in [18] by measuring changes in airway resistance (*R*_*L*_, cmH_2_O/mL/s) and lung dynamic compliance (*C*_dyn_, mL/cmH_2_O) after a challenge with aerosolized methacholine (MCh, 0.78~25 mg/mL) using FlexiVent system (SCIREQ, Montreal, Quebec, Canada). Anesthetized (urethane 600 mg/kg and chloralose 120 mg/kg, i.p.) and tracheostomized (stainless steel cannula, 18 G) mice were mechanically ventilated (150 breaths/min, tidal volume of 10 mL/kg, positive end-expiratory pressure of 3 cmH_2_O); (2) in unrestrained animals, AHR was assessed by barometric plethysmography [19] using a whole-body plethysmograph (WBP) and analyzed using Life Science Suite P3 Analysis Modules (Gould, LDS Test and Measurement LLC, Valley View, OH, USA) software. Mice were placed into the main chamber of the WBP and the baseline enhanced pause (*P*_enh_) value was determined. The mice were then nebulized with phosphate-buffered saline (PBS) and subsequently with increasing doses (6.25~50 mg/mL) of methacholine (MCh) for 3 min per nebulization and readings were made of the breathing parameters 3 min after each nebulization to determine *P*_enh_ values. Twenty-four hours after the *P*_enh_ determination, these mice were anesthetized with pentobarbital (50 mg/kg, i.p.), and lavaged *via* a tracheal tube with PBS (1 × 1.0 mL, 37°C). After lavage, blood was collected from the jugular vein and allowed to coagulate. The collected bronchoalveolar lavage fluid (BALF) and coagulated blood were centrifuged at 630 g for 7 min and at 3700 g for 10 min, respectively, at 4°C. After centrifugation, the supernatants of BALF and serum were stored at −20°C until the determination of cytokines, including interleukin (IL)-2, IL-4, IL-5, tumor necrosis factor (TNF)-*α*, and interferon (IFN)-*γ* by flow cytometric methods [20] using mouse Th1/Th2 cytokine CBA kits, and total immunoglobulin E (IgE) and total IgG_2a_ using enzyme-linked immunosorbent assay (ELISA) kits (Pharmingen, San Diego, CA, USA) according to the recommendations of the manufacturer. The minimum concentrations of cytokine and Ig were 1 pg/mL and 1 ng/mL, respectively. All the undetectable data were taken as 0.5 pg/mL and 0.5 ng/mL, respectively. OVA-specific IgE was measured by a slightly modified version of the method described previously [21]. Wells were coated with 100 *μ*L of OVA (20 *μ*g/mL) instead of the capture antibody. Levels were expressed in arbitrary units, where 1 arbitrary unit equaled the optical density of the sample divided by the optical density of unchallenged mouse serum or BALF (standard). The pellet from BALF was resuspended in ACK lysing buffer (1.658 g NH_4_Cl, 0.2 g KHCO_3_ and 1.44 mg EDTA in 200 mL of water) to lyse the residual erythrocytes in each sample. The number of inflammatory cells was counted using a hemocytometer (Hausser Scientific, Horsham, PA, USA). Cytospun slides were stained and differentiated in a blinded fashion by counting at least 100 cells under light microscopy.

### 2.4. Xylazine/Ketamine-Induced Anesthesia

According to the modified version of the method described by Robichaud et al. [22], biochanin A (30~300 *μ*mol/kg, subcutaneously (s.c.)) or Ro 20-1724 (0.01~1 *μ*mol/kg, s.c.), a reference drug, was injected into 8~12 week-old female BALB/c mice 1 h or 15 min prior to an i.p. injection of xylazine (10 mg/kg)/ketamine (70 mg/kg). The vehicle (control) for biochanin A or for Ro 20-1724 was a mixture of alcohol : DMSO : Tween 20 : saline (0.5 : 0.5 : 1 : 8, v/v), or alcohol : DMSO : PEG 400 : saline (0.5 : 0.5 : 1 : 8, v/v), respectively. The duration of anesthesia was measured from the time of losing the righting reflex (i.e., when a mouse remains on its back no longer spontaneously righting itself to a prone position), until its return [22].

### 2.5. OVA-Induced Tracheal Contractions In Vitro

Male Hartley guinea pigs (500~600 g) were sensitized by intramuscular injections into each thigh of 0.7 mL of 5% (w/v) OVA in saline on days 1, 4, and 43, and in adjuvant on days 25 and 39, according to a modified version of previously described method [23]. Three days after the last injection, sensitized guinea pigs were sacrificed by cervical dislocation, and the tracheas were removed. Each trachea was cut into six segments. Each segment consisted of three cartilage rings. All segments were cut open opposite the trachealis. After the segments were randomized to minimize regional variability, one end was tied to holders via silk sutures, placed in 5 mL of normal Krebs solution containing indomethacin (3 *μ*M), gassed with a mixture of 95% O_2_ plus 5% CO_2_ at 37°C, and the other end of each segment was attached to force displacement transducers (Grass FT03, Quincy, MA, USA) for the isometric recording of tension changes on a polygraph (Gould RS3200, Cleveland, OH, USA). The composition of the normal Krebs solution was (mM): NaCl 118, KCl 4.7, MgSO_4_ 1.2, KH_2_PO_4_ 1.2, CaCl_2_ 2.5, NaHCO_3_ 25, and dextrose 10.1. Tissues were suspended in normal Krebs solution under an initial tension of 1.5 g and allowed to equilibrate for at least 1 h and washed at 15-min intervals. After the tissues were precontracted with KCl (60 mM) and washed with normal Krebs solution, OVA (0.1~100 *μ*g/mL) was cumulatively added, and contractions were allowed to reach a steady state at each concentration. To evaluate the suppressive effect of biochanin A on OVA-induced contractions, each tissue sample was preincubated with concentration (3~30 *μ*M) of biochanin A or its vehicle for 15 min and then again challenged with cumulative OVA. Therefore, log concentration-response curves of OVA were constructed in the absence and presence of biochanin A. The tension of the precontraction induced by KCl was set at 100%.

### 2.6. Statistical Analysis

Concentrations of test compounds producing 50% of the maximum activity (IC_50_ or EC_50_ value) were compared. The IC_50_ and EC_50_ values were calculated employing nonlinear regression analysis using SigmaPlot 10.0 (Sigma Chemical). All values are given as the mean ± SEM. Differences among values were statistically calculated using one-way analysis of variance (ANOVA), and determined by Dunnett's test. The difference between two values, however, was determined by Student's *t*-test. Differences of *P* < .05 were considered statistically significant.

## 3. Results

### 3.1. PDE4_H_/PDE4_L_ Ratios

**Figure 1:**
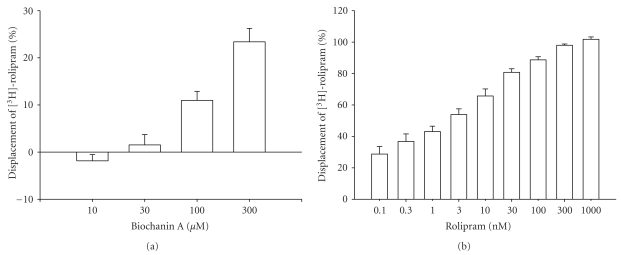
Displacement of [^3^H]-rolipram by biochanin A (a) and rolipram (b) in high-affinity rolipram binding sites of guinea pig whole-brain particulates. Each value represents the mean ± SEM. The numbers of experiments for biochanin A and rolipram were 4 and 8, respectively.

Biochanin A (10~300 *μ*M) concentration-dependently displaced [^3^H]-rolipram binding on HARBSs of guinea pig brain cell membranes (Figure 1(a)). At the highest concentration (300 *μ*M), however, the percentage displacement by biochanin A was 23.4%  ± 2.9% (*n* = 4). The concentration cannot exceed 300 *μ*M owing to the solubility of biochanin A. In other words, the EC_50_ value of biochanin A for the displacement was >300 *μ*M. Rolipram (0.1~1000 nM), a selective PDE4 inhibitor, also concentration-dependently displaced [^3^H]-rolipram binding on HARBSs (Figure 1(b)). In contrast to biochanin A, the percentage of the displacement by rolipram at the highest concentration (1000 nM) was 102%  ± 1.5% (*n* = 8). The EC_50_ value of rolipram for displacement was 5.2 ± 1.9 nM (*n* = 8). As defined in the Methods section, therefore, the PDE4_H_ values of biochanin A and rolipram were >300 *μ*M and 5.2 ± 1.9 nM, respectively. To inhibit PDE4 catalytic activity, the IC_50_ values of biochanin A and rolipram were reportedly required to be 8.5 *μ*M [6] and 2.3 *μ*M [24], respectively. These were adopted as the PDE4_L_ values, because the anti-inflammatory effects of PDE4 inhibitors were reportedly associated with the inhibition of PDE4 catalytic activity [25], and the anti-inflammatory effects were correlated with the inhibition of PDE4_L_ [16]. Thus, PDE4_H_/PDE4_L_ values of biochanin A and rolipram were calculated to be >35 and 0.002, respectively.

### 3.2. Suppression of Airway Hyperresponsiveness In Vivo

**Figure 2:**
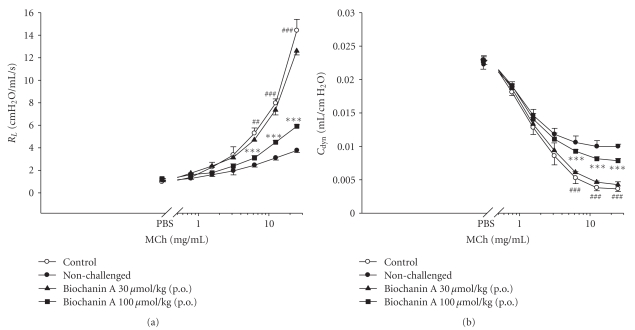
Effects of biochanin A (30~100 *μ*mol/kg, p.o.) on *R*_*L*_ (a) and *C*_dyn_ (b) in sensitized mice receiving aerosolized methacholine (MCh, 0.78~25 mg/mL) 2 days after primary allergen challenge. ^##^ *P* < .01, and ^###^ *P* < .001, compared to the nonchallenged group. ***P < .001, compared to the control (vehicle) group. The number of mice in each group was 10. PBS: phosphate-buffered saline.

*R*_*L*_ values at the baseline for the control (vehicle), nonchallenged, and 30, and 100 *μ*mol/kg biochanin A groups were 0.94 ± 0.14, 1.07 ± 0.03, 1.09 ± 0.05, and 1.26 ± 0.06 cmH_2_O/ml/s, respectively, and these values did not differ significantly. *R*_*L*_ values of PBS nebulization for each group were 0.98 ± 0.16, 1.07 ± 0.03, 1.14 ± 0.07, and 1.26 ± 0.05 cmH_2_O/ml/s, respectively, and again did not differ significantly. Administration of nebulized PBS did not influence the *R*_*L*_ values of the baseline in any group. However, MCh (6.25~25 mg/mL) concentration-dependently and significantly increased *R*_*L*_ values (Figure 2(a)) and decreased *C*_dyn_ values (Figure 2(b)) in the control sensitized and challenged group when compared to the nonchallenged group. Biochanin A 100 *μ*mol/kg (p.o.) significantly suppressed these changes (Figure 2).

**Figure 3:**
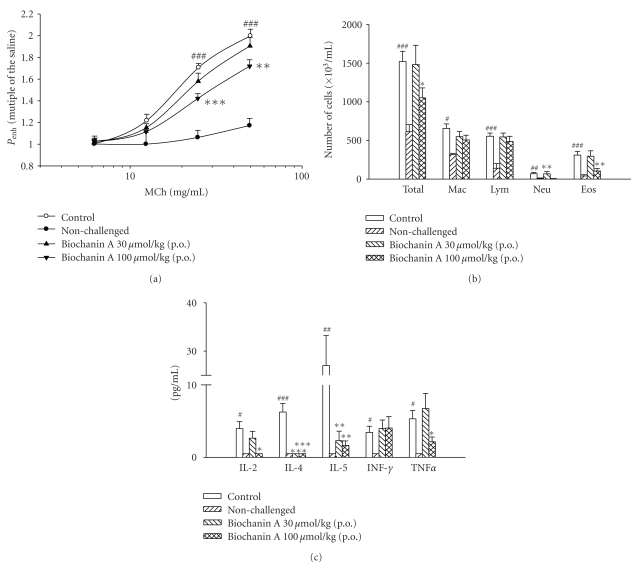
Effects of biochanin A (30~100 *μ*mol/kg, p.o.) on *P*_enh_ (a), inflammatory cells (b), and cytokines (c) in sensitized mice receiving aerosolized methacholine (MCh, 6.25~50 mg/mL) 2 days after primary allergen challenge. ^#^ *P* < .05, ^##^ *P* < .01, and ^###^ *P* < .001, compared to the nonchallenged group. *P < .05, **P < .01, and ***P < .001, compared to the control (vehicle) group. The number of mice in each group was 10. PBS, phosphate-buffered saline; Total, total cells; Mac, macrophages; Lym, lymphocytes; Neu, neutrophils; Eos, eosinophils; IL, interleukin; TNF-*α*, tumor necrosis factor-*α*; TNF-*γ*, tumor necrosis factor-*γ*.

*P*_enh_ values at the baseline for the control (vehicle), nonchallenged, and 30, and 100 *μ*mol/kg biochanin A groups were 2.45 ± 0.05, 2.45 ± 0.03, 2.44 ± 0.10, and 2.50 ± 0.04, respectively, and these values did not differ significantly. *P*_enh_ values of PBS nebulization for each group were 2.40 ± 0.05, 2.46 ± 0.06, 2.40 ± 0.08, and 2.43 ± 0.08, respectively, and again did not differ significantly. Administration of nebulized PBS did not influence the *P*_enh_ value of the baseline in any group. However, MCh (6.25~50 mg/mL) concentration-dependently increased *P*_enh_ values from 1-fold of PBS exposure to 2.00 ± 0.06-fold in control sensitized and challenged mice (Figure 3(a)). *P*_enh_ values of MCh at 25 and 50 mg/mL in control sensitized and challenged mice were significantly enhanced compared to those in nonchallenged mice. Biochanin A (100 *μ*mol/kg, p.o.) significantly attenuated the enhancement of *P*_enh_ values induced by 25 and 50 mg/mL MCh (Figure 3(a)).

### 3.3. Suppression of Inflammatory Cells in BALF

Total inflammatory cells, macrophages, lymphocytes, neutrophils, and eosinophils from the BALF of control-sensitized and challenged mice increased significantly when compared to those of nonchallenged mice (Figure 3(b)). Biochanin A (100 *μ*mol/kg, p.o.) also significantly suppressed the increase in the number of total inflammatory cells, neutrophils, and eosinophils (Figure 3(b)).

### 3.4. Suppression of Cytokines in BALF

Compared to those in nonchallenged mice, levels of cytokines, such as IL-2, IL-4, IL-5, IFN-*γ*, and TNF-*α*, in the BALF of control-sensitized and challenged mice significantly increased (Figure 3(c)). Biochanin A (100 *μ*mol/kg, p.o.) significantly suppressed increases in the levels of IL-2, IL-4, IL-5, and TNF-*α*. Even at a dose of 30 *μ*mol/kg, biochanin A significantly suppressed increases in the levels of IL-4 and IL-5. However, biochanin A at a dose of 30 or 100 *μ*mol/kg did not influence the level of IFN-*γ* compared to the control (Figure 3(c)).

### 3.5. Effects on IgG_2a_ and IgE in the Serum and BALF

**Figure 4:**
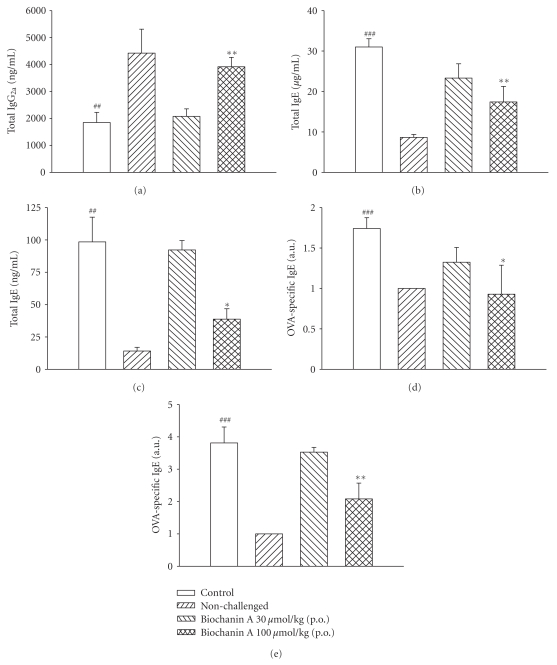
Effects of biochanin A (10~100 *μ*mol/kg, p.o.) on the total IgG_2a_ (a) level in the serum, and total IgE (b, c) and OVA-specific IgE (d, e) levels in the serum (b, d), and BALF (c, e) of sensitized mice receiving aerosolized methacholine (MCh, 6.25~50 mg/mL) 2 days after primary allergen challenge. ^##^ *P* < .01, and ^###^ *P* < .001, compared to the nonchallenged group. *P < .05, and **P < .01, compared to the control (vehicle) group. Each value represents the mean ± SEM. The number of mice in each group was 10.

The total IgG_2a_ level in the serum of control sensitized and challenged mice was significantly reduced compared to that of nonchallenged mice. Biochanin A (100 *μ*mol/kg, p.o.) significantly reversed this reduction (Figure 4(a)). Levels of total and OVA-specific IgE in the serum and BALF for control sensitized and challenged mice were significantly enhanced compared to those of nonchallenged mice. Biochanin A (100 *μ*mol/kg, p.o.) significantly suppressed these enhancements (Figures 4(b)–4(e)).

### 3.6. No Effect on Xylazine/Ketamine-Induced Anesthesia

**Figure 5:**
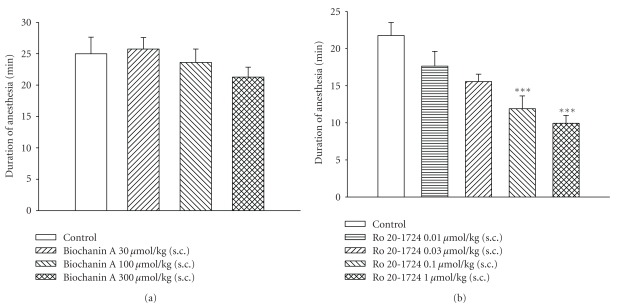
Effects of subcutaneously administered (a) biochanin A and Ro 20-1724 (b) on the duration of xylazine (10 mg/kg, i.p.)/ketamine (70 mg/kg, i.p.)-induced anesthesia in mice. Biochanin A or Ro 20-1724 was administered 15 min or 1 h prior to anesthesia, respectively. ***P < .001, compared to the vehicle (control). Each value represents the mean ± SEM. The numbers of mice used for biochanin A and Ro 20-1724 were 14 and 21, respectively.

The durations of xylazine/ketamine-induced anesthesia in control (vehicle) mice of the biochanin A- and Ro 20-1724-treated groups were 25.0 ± 2.7 (*n* = 13) and 21.8 ± 1.7 min (*n* = 21), respectively. Biochanin A (30~300 *μ*mol/kg, s.c.) did not significantly affect the duration (Figure 5(a)). However, Ro 20-1724 (0.01~1 *μ*mol/kg, s.c.) dose-dependently shortened the duration, and at doses of 0.1 and 1 *μ*mol/kg (s.c.) significantly shortened the duration (Figure 5(b)).

### 3.7. Inhibition of OVA-Induced Contractions In Vitro

**Figure 6:**
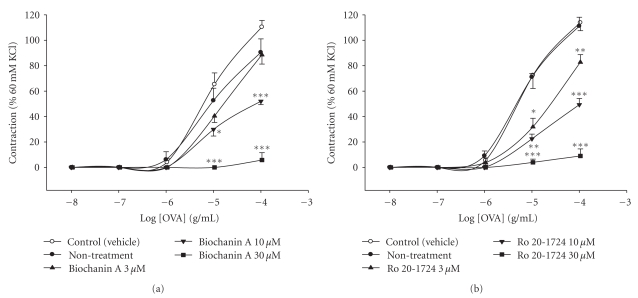
Effects of biochanin A (a) and Ro 20-1724 (b) on cumulative ovalbumin (OVA)-induced contractions in isolated sensitized guinea pig trachealis. *P < .05, **P < .01, and ***P < .001, compared to the control (vehicle). Each value represents the mean ± SEM (*n* = 5 ~ 8).

For sensitized guinea pig trachea in isolation, 60 mM of KCl evoked contractions and increased tension to 968 ± 35 mg (*n* = 54) that was set to 100%. OVA (0.01~100 *μ*g/mL) concentration-dependently enhanced the tension from the baseline to 110.4% ± 5.2% (*n* = 8) of the 60 mM KCl-induced contractions (Figure 6(a)). The log concentration-response curve of OVA was unaltered by 1 *μ*M nifedipine (data not shown), a selective voltage-dependent calcium channel blocker [26], although it significantly relaxed 194 ± 67 mg (*n* = 3) from the baseline tension after a 15-min preincubation at this concentration. Biochanin A (10 and 30 *μ*M) concentration-dependently and significantly relaxed respective 217 ± 44 mg (*n* = 6) and 416 ± 76 mg (*n* = 6) from the baseline tension, and inhibited OVA (10~100 *μ*g/mL)-induced contractions (Figure 6(a)). Similarly, Ro 20-1724 (3, 10, and 30 *μ*M) concentration-dependently and significantly relaxed respective 297 ± 83 mg (*n* = 5), 434 ± 109 mg (*n* = 5) and 731 ± 91 mg (*n* = 5) from the baseline tension, and inhibited OVA (10~100 *μ*g/mL)-induced contractions (Figure 6(b)). The IC_50_ value of biochanin A for inhibiting OVA (100 *μ*g/mL)-induced contractions was calculated as 8.1 ± 0.8 *μ*M (*n* = 5).

## 4. Discussion

Allergic asthma is a chronic respiratory disease characterized by AHR, mucus hypersecretion, bronchial inflammation, and elevated IgE levels. T-helper type 2 (Th2) cells, together with other inflammatory cells such as eosinophils, B cells, and mast cells were proposed as critical to the initiation, development, and chronicity of this disease [27]. One hypothesis emphasized an imbalance in Th cell populations favoring the expression of Th2 over Th1 cells. Cytokines released from Th2 cells are IL-4, IL-5, IL-6, IL-9, and IL-13, and those from Th1 cells are IL-2, IL-12, IFN-*γ*, and TNF-*α* [28, 29]. Although the solubility and absorption of biochanin A, an isoflavone, is poor, biochanin A (100 *μ*mol/kg, p.o.) was observed to significantly decrease *R*_*L*_ (Figure 2(a)), increase *C*_dyn_ (Figure 2(b)) and reduce *P*_enh_ values (Figure 3(a)) suggesting that it significantly suppresses AHR. It also suppressed the number of total inflammatory cells, neutrophils, and eosinophils in the BALF of sensitized and challenged mice (Figure 3(b)). The reason that macrophages and lymphocytes were not influenced by biochanin A at this dose is unclear. Biochanin A (100 *μ*mol/kg, p.o.) also suppressed levels of IL-2, IL-4, IL-5, and TNF-*α*. Biochanin A even at 30 *μ*mol/kg (p.o.) significantly suppressed levels of IL-4, and IL-5, although it did not influence all types of inflammatory cells at this dose. However, the level of IFN-*γ* was unaffected by 30 or 100 *μ*mol/kg (p.o.) biochanin A. These results suggest that biochanin A suppresses both Th2 and Th1 cells that were implicated in autoimmune and atopic diseases, respectively [30]. On the other hand, biochanin A was reported to enhance IL-4 production in activated T cells through two independent pathways [31]. This* in vitro* result is inconsistent with our present *in vivo* results, but the reason is unclear.

IL-4 and IL-13 were shown to induce AHR in mouse asthma models [32, 33]. IL-4 has three primary effects. First, IL-4 promotes B cell differentiation to plasma cells secreting antigen-specific IgE antibodies. Second, IL-4 promotes mast cell proliferation. Third, increased IL-4 upregulates endothelial cell expression of adhesion molecules for eosinophils [34]. IL-5 mobilizes and activates eosinophils, leading to a release of major basic proteins, cysteinyl-leukotrienes, and eosinophil peroxidase that contribute to tissue damage and AHR [33, 35]. Phosphoinositide 3-kinase *δ* (p110*δ*) was shown to play a crucial role in the development, differentiation, and antigen receptor-induced proliferation of mature B cells [36, 37]. Inhibition of p110*δ* attenuates allergic inflammation in airways and AHR in a murine asthma model [37, 38]. In addition, IL-4 and IL-13 are important in directing B cell growth, differentiation, and the secretion of IgE [39]. However, IFN-*γ* released from Th1 cells preferentially directs B cell switching of IgM to IgG_2a_ and IgG_3_ in mice [40, 41]. The biological activities of IgE are mediated through the high-affinity IgE receptor (Fc*ε*RI) on mast cells and basophils. Cross-linking of the Fc*ε*RI initiates multiple signal cascades leading to cellular degranulation and activation [42, 43]. The activity of p110*δ* was reported to be critical for allergen-IgE-induced mast cell degranulation and the release of cytokines [44]. Inhibition of p110*δ* therefore attenuates the production of IgE as well as allergen-IgE-induced mast cell activation during allergic inflammation. It was suggested that calcium channels in mast cell membranes differ from those in cardiovascular tissues [45], which are sensitive to nifedipine. In the present *in vitro *results, nifedipine (1 *μ*M) significantly relaxed the baseline tension but did not influence the cumulative OVA-induced contractions of isolated sensitized guinea pig trachealis, suggesting that nifedipine did not inhibit degranulation of mast cells [46], because nifedipine has no anti-inflammatory effects. In contrast, biochanin A (10~30 *μ*M) significantly relaxed the baseline tension and inhibited cumulative OVA-induced contractions in isolated sensitized guinea pig trachealis, suggesting that biochanin A inhibits degranulation of mast cells and, at least partially, prevents inflammation. This inhibition is unrelated to its relaxant effects on smooth muscle. Although biochanin A possesses both smooth muscle relaxant and anti-inflammatory effects, it inhibits cumulative OVA-induced contractions in isolated sensitized guinea pig trachealis due mainly to its anti-inflammatory effects. The IC_50_ value of biochanin A for inhibiting OVA (100 *μ*g/mL)-induced contractions was calculated to be 8.1 *μ*M, and is similar to that (8.5 *μ*M) for inhibiting PDE4 activity [6]. In addition, biochanin A (100 *μ*mol/kg, p.o.) dose-dependently and significantly suppressed total and OVA-specific IgE levels in the serum and BALF, and enhanced the level of total IgG_2a_ in the serum of sensitized and challenged mice, suggesting that biochanin A has immunoregulatory effects.

Selective PDE4 inhibitors specifically prevent the hydrolysis of cAMP, a 3′,5′-cyclic nucleotide, and therefore have broad anti-inflammatory effects such as the inhibition of cell trafficking and of cytokine and chemokine release from inflammatory cells. The second-generation PDE4 inhibitors, cilomilast and roflumilast have reached the clinical trial stage and exhibited a number of beneficial effects for treating asthma and COPD [47]. The effectiveness of these PDE4 inhibitors may be limited by their clinical potency when using doses that have minimal adverse effects on headaches, diarrhea, nausea, and abdominal pain. The PDE4_H_/PDE4_L_ ratios of cilomilast and roflumilast were reported to be 117.8 nM/120 nM (1), and 2.4 nM/0.8 nM (3), respectively [15, 48], and are considerably greater than those (0.01~0.001) of rolipram [16]. Due to its adverse effects or lack of efficacy, cilomilast was discontinued for asthma treatment after phase II clinical trials in 2003 [47]. In terms of tolerability over 6 months with 15 mg twice daily for COPD in a phase III study, cilomilast was found to be associated with higher frequencies of diarrhea and nausea than with a placebo [47]. Roflumilast is still being evaluated for asthma and COPD in phase III clinical trials at present, and is reported to reduce those adverse effects after longer-term treatment at 0.5 mg once daily [47]. Recently, roflumilast was reported to significantly improve mean pre- or postbronchodilator forced expiratory volume for 1 s (FEV_1_) in patients with moderate-to-severe COPD, compared to a placebo. However, nausea, diarrhea, weight loss, and headache were more frequent in patients in the roflumilast group. These adverse events were associated with an increase in patient withdrawal [49, 50]. The PDE4_H_/PDE4_L_ ratio of AWD 12-281, another selective PDE4 inhibitor, was reported to be 104 nM/9.7 nM (approximately 11) [13]. AWD 12-281 has been undergoing clinical development phase IIa trials for COPD, and has been reported as a potentially unique drug for the topical treatment of asthma and COPD [51]. AWD 12-281 was reported as a very promising drug for treating lung inflammation when administered by inhalation and for treating atopic dermatitis [52]. However, AWD-12-281 was also discontinued in clinical trials of both asthma and COPD owing to its lack of efficacy [53, 54]. Many compounds are in development but have yet to reach the market as a monotherapy, and will remain so until the emetic liability has been reduced. However, inhaled GSK256066 has demonstrated efficacy in trials for asthma [55] and an oral apremilast was reported to be clinically effective for treating severe plaque-type psoriasis [56]. Another strategy for developing new PDE4 inhibitors may consider PDE4 subtypes (A~D). PDE4D inhibition in nontarget tissues promotes emesis, because the PDE4D knock-out mice showed a reduction in anesthesia triggered by xylazine/ketamine, which is used as a surrogate marker for emesis in mice, a nonvomiting species [22]. Recently, small-molecule allosteric modulators of PDE4D that do not completely inhibit enzymatic activity were reported to reduce emesis and to have therapeutic benefit for brain distribution, such as Alzheimer's disease, Huntington's disease, schizophrenia, and depression [57]. In contrast, selective inhibition of PDE4A and/or PDE4B in pro-inflammatory and immune cells is believed to evoke the therapeutically desired effects of these drugs [58]. Compared to PDE4A and PDE4B, cilomilast has a higher potency for PDE4D, while roflumilast is nonselective for these four PDE4 subtypes with a similar degree of inhibition [59]. No research into the role of AWD 12-281 in the inhibition of PDE4 subtypes has been conducted. Whether Biochanin A selectively inhibits PDE4 subtype requires further investigation. The increased cAMP levels induced by these selective PDE4 inhibitors activate cAMP-dependent protein kinase which may phosphorylate and inhibit myosin light-chain kinase, thus inhibiting contractions [60]. The precise mechanism by which relaxation is produced by this second-messenger pathway is unknown, but it may result from a decrease in intracellular Ca^2+^ ([Ca^2+^]_i_). The decrease in [Ca^2+^]_i_ may be due to a reduction in the influx of Ca^2+^, enhanced Ca^2+^ uptake into the sarcoplasmic reticula, or enhanced Ca^2+^ extrusion through the cell membrane [60]. Biochanin A was reported to relax isolated rabbit basilar arteries by a mechanism of Ca^2+^ entry blockade [61]. Because biochanin A is a selective PDE4 inhibitor [6], this compound should have the effect of decreasing [Ca^2+^]_i_ and a relaxant effect in isolated guinea pig trachea. Consequently, red clover has been reported to have an antispasmodic effect in previous literature [3]. In contrast to animals, a single dose of cilomilast [62] or roflumilast [63] per day was reported to have no bronchodilator effect on humans. Whether biochanin A has bronchodilator effect requires investigation in randomized, placebo-controlled clinical studies.

Following oral administration of biochanin A, genistein, a demethylated metabolite of biochanin A, may have been produced. Biochanin A and genistein have been reported to inhibit protein tyrosine kinase (PTK) of epidermal growth factor receptor with IC_50_ values of 91.5 *μ*M and 2.6 *μ*M, respectively [64]. In a guinea pig model of asthma, genistein was reported to have anti-inflammatory effects in airway *via* PTK inhibition [65]. Moreover, an increase in the consumption of soy genistein was reported to be associated with improved lung function in patients with asthma [66]. In clinical trials, compared to subjects receiving a placebo, subjects receiving genistein (54 mg/day for 2 years) revealed a significantly (19% versus 8%, *P* = .002) higher incidence of discontinued therapy following adverse gastrointestinal events, such as abdominal pain, epigastric pain, dyspepsia, and constipation [67]. In a murine model of allergic asthma, we also reported that genistein concentration-dependently inhibited 2 nM [^3^H]-rolipram bound to HARBSs of brain cell membranes and shortened xylazine/ketamine-induced anesthesia at a dose level of 100 *μ*mol/kg (s.c.), suggesting that higher doses of genistein may induce adverse gastrointestinal side effects [68]. However, in the present results, biochanin A, even at 300 *μ*mol/kg (s.c.), did not shorten this kind of anesthesia. This suggests that biochanin A, 1 h after injection (s.c.), is demethylated slightly to form genistein. In the present *in vivo* study, biochanin A (100 *μ*mol/kg, p.o.) significantly enhanced the total serum IgG_2a_ levels, which had probably switched from IgM due to an increase in IFN-*γ* level [40, 41]. The increased IFN-*γ* levels may have been offset by genistein, which has recently been reported to inhibit IFN-*γ* levels [68], when biochanin A is demethylated. These results suggest that 24~50 h after oral administration, biochanin A is demethylated to produce genistein. Thus, long-term use of biochanin A may cause adverse gastrointestinal effects, such as abdominal pain, epigastric pain, dyspepsia, and constipation, particularly at higher doses. However, accurately determining the concentration of biochanin A and the demethylated metabolites in the blood requires further pharmacokinetic investigation.

In the present results, the PDE4_H_ value of biochanin A was >300 *μ*M, suggesting that it had a low affinity for HARBSs of brain cell membranes. Thus the PDE4_H_/PDE4_L_ ratio of biochanin A was >35, which was considerably greater than that of AWD 12-281. In addition, biochanin A did not influence xylazine/ketamine-induced anesthesia. These results are consistent with the low affinity of biochanin A for HARBSs of brain cell membranes. However, Ro 20-1724, a selective PDE4 inhibitor, reversed the anesthetic effects. This reversal may have occurred through presynaptic *α*_2_-adrenoceptor inhibition [69], because MK-912, an *α*_2_-adrenoceptor antagonist, has been reported to reverse xylazine/ketamine-induced anesthesia in rats [70] and trigger vomiting in ferrets [69]. In contrast, clonidine, an *α*_2_-adrenoceptor agonist, prevented emesis induced by PDE4 inhibitors in ferrets [69]. In the present results, the fact that biochanin A did not reverse the duration of xylazine/ketamine-induced anesthesia may have been due to a lack of genistein demethylated from biochanin A.

**Figure 7:**
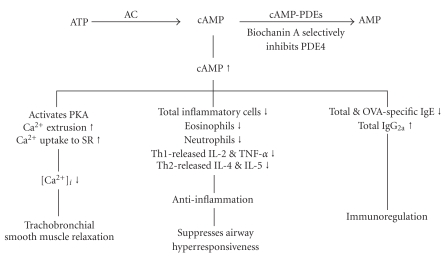
Mechanisms involved in the action of Biochanin A. Biochanin A selectively inhibits phosphodiesterase (PDE)4 activity resulting in an increase in cAMP, activating cAMP dependent protein kinase (PKA) and increasing calcium extrusion from the intracellular space and uptake to sarcoplasmic reticula (SR). Biochanin A largely decreased the concentration of intracellular calcium ([Ca^2+^]_i_) resulting in trachobronchial smooth muscle relaxation. The increase in cAMP also had anti-inflammatory and immunoregulatory effects. AC, adenylate cyclase; Th, T-helper cells; Ig, immunoglobulin; IL, interleukin; TNF-*α*, tumor necrosis factor-*α*. Up and down arrows indicate increases and decreases, respectively.

In conclusion, the present results support the traditional use of red clover containing biochanin A for the treatment of allergic asthma and COPD, despite evidence of adverse gastrointestinal effects following long time use. The mechanisms of biochanin A are summarized in Figure 7.

## Abbreviations

AHR: airway hyperresponsiveness
BALF: bronchoalveolar lavage fluid
cAMP: adenosine 3′,5′ cyclic monophosphate
cGMP: guanosine 3′,5′ cyclic monophosphate
COPD: chronic obstructive pulmonary disease
DMSO: dimethyl sulfoxide
EDTA: ethylenediaminetetraacetic acid
HARBSs: high-affinity rolipram binding sites
IFN-*γ*: interferon-*γ*
Ig: immunoglobulin
IL: interleukin
*K*_*i*_: dissociation constant for inhibitor binding
MCh: methacholine
OVA: ovalbumin
PBS: phosphate-buffered saline
PDE: phosphodiesterase
PDE4_H_: high affinity for PDE4
PDE4_L_: low affinity for PDE4
PEG: polyethyleneglycol
*P*_enh_: enhanced pause
PMSF: phenylmethanesulfonyl fluoride
Ro 20-1724: 4-(3-butoxy-4-methoxybenzyl)-2-imidazolidinone
Th: T-helper
TNF-*α*: tumor necrosis factor-*α*.
